# Informal caregivers’ perspectives on caring for elderly people in Romania: a qualitative study

**DOI:** 10.1186/s12913-026-14085-1

**Published:** 2026-01-23

**Authors:** Daniel Adrian Lungu, Alina Ionescu-Corbu, Francesca Pennucci, Florin Tibu

**Affiliations:** 1 SHARE, Centre for Resilience in Healthcare, Department of Quality and Health Technology, Faculty of Health Sciences, University of Stavanger, Stavanger, Norway; 2 Faculty of Educational Sciences, Stefan cel Mare University of Suceava, Suceava, Romania; 3 Interdisciplinary Research Center “Health Science”, Scuola Superiore Sant’Anna, Pisa, Italy; 4 Department of Biomedical Sciences, Stefan cel Mare University of Suceava, Suceava, Romania

**Keywords:** Long-term care, Informal caregivers, Elderly care, Romania

## Abstract

**Background:**

Informal caregiving is a critical component of elderly care, particularly in countries like Romania where formal long-term care (LTC) services are underdeveloped or inaccessible to many. Family caregivers often provide the majority of daily care, yet little is known about how they perceive this role - whether it is taken on voluntarily, out of love, as an unavoidable necessity, a combination of the abovementioned, or out of other reasons. This study explores Romanian caregivers’ perspectives on the motivations behind caregiving, their lived experiences, and their preferences and expectations regarding future care arrangements, especially in the context of a healthcare system that does not formally recognise or support their contributions.

**Methods:**

A qualitative study was conducted using in-depth semi-structured interviews with 14 informal caregivers, primarily family members caring for elderly relatives. Participants were purposively recruited to reflect a diversity of caregiving contexts (e.g. spouses, adult children, grandchildren). The interviews explored motivations, challenges, rewards, and perceptions of formal support systems and LTC alternatives. Transcripts were analysed in the original Romanian language using inductive thematic analysis, with emergent themes later translated into English for reporting.

**Results:**

Caregivers described a continuum between obligation and choice. Many viewed caregiving as a moral responsibility driven by family values or reciprocity, while others emphasized their personal willingness and affection, reporting they cared “with no feeling of obligation, only love.” Participants often reported burdens including emotional and physical exhaustion, financial strain, social isolation, and limited access to formal support. Despite these challenges, caregivers also expressed emotional rewards such as fulfillment, pride, and strengthened family bonds. Most preferred home-based care over institutional care, citing cultural norms, cost, and mistrust of facilities. At the same time, caregivers voiced strong needs for more structured support: accessible information, formal training, and inclusion by healthcare professionals.

**Conclusions:**

Informal caregivers in Romania play an indispensable role in supporting the elderly, often with minimal formal assistance. Their work is driven by both love and obligation and marked by both burdens and rewards. Findings point to the urgent need for policy reforms that recognise informal caregivers and provide structured support - through financial, educational, and psychosocial interventions -within a more integrated and culturally responsive LTC system.

**Supplementary Information:**

The online version contains supplementary material available at 10.1186/s12913-026-14085-1.

## Background

According to the OECD, LTC comprises a mix of personal care, nursing care, and supportive services provided to individuals who, for an extended period, are dependent on help with activities of daily living [1]. Family caregivers play a key role in long-term care (LTC) provision worldwide. Estimates suggest that as much as 80% of LTC in Europe is provided by informal (unpaid) carers [2]. Exploring motivations underlying LTC sustained by informal caregivers might become more and more important as life expectancy throughout Europe is increasing, with a tendency to spending more years in poor health [3]. Across European countries, between about 10% and 25% of the population engages in informal caregiving, depending on whether informal caregiving is defined as providing care to co-residents only or also to non co-residents ​ [2, 4]. These caregivers – often middle-aged or older women caring for aging parents or spouses – form the “basis” of LTC systems [2, 5].

Informal care is widely seen as a cost-effective way to enable older people to remain at home and avoid institutionalisation​. However, this reliance on family care comes at a cost: caregivers may face impacts on their employment, health, and well-being​ [2, 6]. In recent years, there is growing recognition that supporting informal carers is essential for sustainable LTC, as reflected in European policies that call for providing informal caregivers with training, counselling, and financial support​ [7].

In this article, we use the term LTC to refer specifically to care provided to older adults (65+) living at home or in community settings (e.g. assisted living or sheltered living arrangements, day care centers for older adults, non-residential facilities managed by municipalities, NGOs, or faith-based organisations) in Romania.

Romania registers some of the highest rates of reported LTC needs for the 65 + age group population - more than 50% reporting needing help with at least one personal care activity, compared to an EU average of 30% - [3] and this tendency was observed in some recent studies suggesting that the number of patients accompanied by their families in hospitals increased to almost 80% in the last few years [8]. Thus, Romania represents an extreme case of an LTC system dependent on informal care. Public provision of formal LTC services in Romania is very limited, and responsibility for elderly care has traditionally fallen on the family​. Fewer than 0.5% of older Romanians are in residential care facilities, one of the lowest institutionalisation rates in Europe​ [9].

There are no distinct insurance programmes targeting LTC, and while there are some social protection options for elderly people with a certain degree of disability (e.g. recognition of disability certificates, access to day-care centres, home-care services, and adaptation of living environment) these provisions are fragmented [3]. Informal caregivers are often responsible for the daily care of patients, from providing basic needs such as washing and feeding, to more complex needs such as providing emotional support and maintaining the relationship with medical care facilities. These responsibilities add to the burden of care, leading to intense suffering, physical exhaustion and ignoring personal needs [10]. This burden is added to the lack of financial support and information that are characteristic to Romania’s healthcare system [11]. Home care and community services are underdeveloped, leaving many care needs unmet by formal providers. Consequently, most assistance for frail older adults is provided by family members or other informal helpers. Informal carers in Romania receive virtually no systematic support or recognition from the state​ [9].

There is no legal status for family caregivers and no dedicated caregiver allowance for elder care​ [12], and until recently, older people and their carers were generally not eligible for cash benefits or other forms of support. A law passed in 2022 introduced the possibility, if a set of criteria is met (caring for a relative or household member with a “serious medical condition”, defined as a condition that severely limits basic activities), for informal caregivers to obtain a reduced number of working hours from their employer, and in some cases, even a financial indemnity. In the case of carers of disabled people, they entitled to an indemnity that depends on a number of factors, i.e. the degree of disability. Data on informal care is scarce and there is some variability within different sources. While a recent report shows that less than 10% of the population provided informal care [3], according to the 2016 European Quality of Life Survey, 16% of Romania’s population reported providing care to an older, disabled, or infirm person several days a week or daily​ [13]. This substantial contribution occurs with minimal public assistance or specific quality standards.

Cultural expectations in Romania strongly favour family caregiving. In a Eurobarometer survey, 56% of Romanian respondents indicated that the best care option for an elderly person living alone would be to live with one of their children​ [5]. It is commonly expected that adult children (especially daughters) or close kin will take care of dependent elderly family members​ [14–16]. Indeed, most informal carers in Romania are women and they provide the most intense care, with more than 20 h per week. According to the OECD figures, 71% of Romanian informal caregivers are women, compared to an average of 60% across OECD countries [17]. They are typically wives caring for spouses or daughters caring for aging parents​ [3]. This familialist norm, combined with the lack of affordable alternatives, can make caregiving feel less like a personal choice and more like an obligatory role one enters by necessity. At the same time, many Romanians view caring for parents as a moral duty and a way to reciprocate the support they once received [18].

Informal caregiving can be rewarding, but international studies consistently show it often imposes profound emotional, physical, and financial hardships on caregivers [4, 19]. They report suffering stress, depression, social isolation, and declines in their own health as a result of their caregiving role [19, 20]. Many also face serious financial strain, as care duties incur out-of-pocket costs and conflict with paid employment – a substantial proportion of family caregivers must reduce work hours or even leave the workforce to meet care demands [19], resulting in lost income and missed career opportunities [21].

Research from across the world highlights that caregivers commonly describe feeling overwhelmed by the burden of responsibilities and the lack of resources or respite available to them [19, 22]. Overall, a large body of qualitative and quantitative evidence from diverse countries has established that informal caregiving for older adults often exacts an emotional, physical, and economic toll on caregivers, exacerbated by insufficient formal support and gendered social norms [23–26]. These internationally consistent findings set the stage for examining contexts where research remains scarce – for instance, there is limited empirical evidence on the experiences of informal caregivers in Romania, representing a notable gap in the literature that this study will address. Quantitative studies highlight the heavy burden on Romanian caregivers, who often need to manage informal care with employment without external help​ [9, 12]. However, there is very little qualitative evidence about how caregivers in Romania perceive their role and cope with daily challenges.

To address this gap, this study qualitatively explores the experiences of informal caregivers of elderly adults (65+) in Romania. We focus on three sub-aims: (1) whether caregivers perceive taking on care as a voluntary choice, an unavoidable necessity, or a combination of different reasons (and the influences behind this perception), (3) caregivers’ lived experiences – including challenges, stresses, and any personal rewards or satisfactions gained from caregiving – and (3) caregivers’ preferences and expectations regarding LTC arrangements in Romania, such as attitudes toward institutional care versus home care and desired support from services. By examining these issues through in-depth interviews, we aim to provide a nuanced understanding of the caregiving experience in the Romanian context. Such insights can inform the development of more responsive policies and services, ensuring that informal caregivers are supported and that elderly care models better align with families’ needs and preferences.

## Methods

### Design and participants

We conducted a qualitative study using semi-structured interviews to gain in-depth insights into the perspectives of Romanian informal caregivers. A purposive sampling strategy was employed to recruit caregivers of older adults (aged ≥ 65) with varying backgrounds and caregiving contexts. Recruitment took place in the context of the Support4Resilience project, a Horizon Europe initiative aimed at improving the mental wellbeing of elderly care workers, leaders, and informal caregivers across several European countries. Participants were approached and recruited by one project member when they accompanied their loved ones to the University Hospital in Suceava (north-eastern Romania) for medical investigations. Participants were interviewed in the period December 2024 - April 2025. Inclusion criteria were that participants be the primary informal caregiver to an older adult (family member or close acquaintance who is the main contact person for the elderly), to not be paid for this task, and able to give informed consent for an interview. We aimed for diversity in gender, age, and relationship to the care recipient to capture a range of experiences.

A total of 14 caregivers (12 women and 2 men) were recruited. Participants ranged in age from mid-20s to 70s. Their caregiving relationships included spouses caring for a partner (*n* = 1), adult daughters or sons caring for one of their parents (*n* = 7) or parent-in-law (*n* = 2), grandchildren caring for grandparents (*n* = 3), and neighbours (*n* = 1). The care recipients suffered from various age-related conditions (such as stroke, dementia, cardiovascular disease, or general frailty), requiring assistance with daily living. Prior to data collection, participants provided written informed consent and were assured of confidentiality and the voluntary nature of participation. An overview of participants’ characteristics is provided in Table 1 below.

**Table 1:** Unique ID, gender, and relationship to the person cared for of the study participants

Participant ID	Gender	Person cared for
P1	Female	Father
P2	Male	Father
P3	Female	Currently father, used to care for the mother as well
P4	Female	Mother
P5	Female	Father
P6	Male	Mother
P7	Female	Grandfather
P8	Female	Father-in-law
P9	Female	Mother
P10	Female	Grandmother
P11	Female	Husband
P12	Female	Neighbour
P13	Female	Grandmother
P14	Female	Father-in-law

### Data collection

Interviews were conducted in Romanian using a semi-structured guide developed by the researchers to address the study’s objectives (Supplementary file S1). The guide is shared with the other empirical partners in the Support4Resilience project, and it will allow comparisons between countries in the future. The interview guide was developed collaboratively by an international group of researchers by combining theories of individual resilience, organisational resilience, and mental well-being that are the backbone of the Support4Resilience project. The guide was first developed in English and subsequently translated to Romanian. A back translation was performed as well by experienced bilingual researchers to ensure the quality of translation. The interview guide covered four main topics: (1) the circumstances and motivations surrounding how the individual became a caregiver (e.g. reasons for the choice); (2) the caregiver’s daily experience and routines, including typical care tasks, challenges faced (physical, emotional, financial, social), and coping strategies; (3) the perceived rewards or positive aspects of caregiving, if any (such as personal growth, satisfaction, improved relationship with the care recipient); and (4) views on LTC and support, including preferences for future care arrangements (e.g. family care versus nursing home care) and expectations of support from healthcare or social services. Follow-up questions were used to elicit concrete examples (e.g. “Can you describe a situation that felt especially difficult/rewarding?”) and to clarify participants’ feelings about their role.

Two trained interviewers (both native Romanian speakers and experienced in qualitative methods) conducted the interviews in person. Interviews lasted between 31 and 90 min, with a mean duration of 51 min. With participants’ permission, each interview was audio-recorded and later transcribed verbatim in Romanian. Field notes were made after each interview to capture contextual observations. Transcripts were anonymised by assigning each participant a code (P1 through P14) and removing any names or identifying details. Participants were informed that they have the opportunity to access, edit, or delete their transcript at any time.

### Data analysis

We analysed the interview data using thematic analysis, following the approach of Braun and Clarke [27] to identify patterns and themes in an inductive, data-driven manner. We selected Braun and Clarke’s reflexive thematic analysis because it provides a flexible yet rigorous framework for identifying patterns of meaning in participants’ accounts. Although originally developed in psychology, the approach is now widely used across health, ageing, and social care research, where it is valued for its theoretical flexibility and suitability for exploratory questions [28, 29]. Our research aimed to understand how informal caregivers interpret their caregiving role, a task that requires an inductive, interpretive approach grounded in participants’ lived experiences. Reflexive thematic analysis supports this goal by allowing systematic coding while enabling researcher reflexivity and sensitivity to cultural context [30].

The analysis was carried out using the original transcripts in Romanian to ensure that nuance and meaning were preserved during coding. In the first phase, two researchers independently read all transcripts in full to achieve immersion and noted initial impressions of the concepts covered. During this phase, reflexive notes and analytic memos were written to capture early impressions, potential concepts, and thoughts about how participants constructed their caregiving experiences (Braun & Clarke step 1: familiarisation with the data). Then one researcher performed open coding on a subset of transcripts to develop a preliminary codebook of recurring concepts. Segments of text that appeared meaningful or relevant to the research aims were labelled with concise codes. At this stage, coding was intentionally broad and inclusive, capturing both semantic and latent meanings. Codes were revised and refined as the researcher moved back and forth between transcripts (Braun & Clarke step 2: generating initial codes). Codes represented ideas such as *“family duty”*,* “no alternative”*,* “physical strain”*,* “emotional stress”*,* “rewarding aspects”*,* “respite needs”*, and so on, corresponding to segments of text. Then the preliminary codebook was shared with the second researcher, and it was discussed to refine code definitions, achieving a consistent coding scheme.

Using the agreed codebook, transcripts were coded systematically with the aid of qualitative analysis software (NVivo 15). New codes were added if salient new ideas emerged in later interviews. After coding, codes were examined and grouped into themes and sub-themes reflecting the research questions. For example, codes related to feeling *“obliged by culture”* or *“it’s my duty”* were grouped under a theme about *Caregiving as duty vs. choice*. Themes were not simply summaries of codes but were constructed to represent patterns of shared meaning across participants’ accounts (Braun & Clarke step 3: constructing initial themes).

Then, candidate themes were reviewed in relation to the coded data and the entire dataset. Some themes were collapsed, split, renamed, or re-organised to ensure they captured a coherent and meaningful story about the data. During this stage, transcripts were revisited repeatedly to check that themes were grounded in participants’ narratives (Braun & Clarke step 4: reviewing and refining themes).

Each theme was refined to articulate its central organising concept - the core idea that captured what the theme was about and how it contributed to understanding caregivers’ experiences. Sub-themes were developed where appropriate to capture nuance within larger themes. Clear names and definitions were written to reflect the essence of each theme and to distinguish them from one another (Braun & Clarke step 5: defining and naming themes).

In the final phase, themes were woven into an analytic narrative supported by vivid data extracts. This write-up went beyond description by interpreting how participants made sense of caregiving, choice, duty, and long-term care within the Romanian context. The reporting was guided by Braun & Clarke’s recommendations for high-quality reflexive thematic analysis (step 6: producing the report).

A total of five major themes emerged, which aligned well with the study’s three focal areas of investigation (perceptions of duty/choice, caregiving experiences, and LTC preferences) while providing more granular insights within each area. A complete overview of the themes and codes is available in Supplementary file S2.

To prepare results for publication, key quotations that exemplified each theme were translated from Romanian to English. Care was taken to preserve the meaning and tone of participants’ words in translation. The translation was reviewed by the Romanian team members to ensure accuracy. Quotations are presented in the results to illustrate the themes, followed by the participant’s code (e.g., P5 for Participant 5).

### Ethical considerations

This study was approved by the Research Ethics Committees of the Stefan cel Mare University of Suceava (approval letter no. 220/25.07.2024) and the Sfantul Ioan cel Nou Clinical Emergency Hospital of Suceava (no. 36/30.07.2024). Participants received written and verbal information about the study’s purpose and what participation entailed. It was emphasised that involvement was voluntary and independent of any medical or social services they and the persons they cared for receive. Informed consent was obtained prior to interviews. Participants were assured that their identities and any personally identifying details would remain confidential. Audio recordings and transcripts were stored securely and accessible only to the research team. In presenting the results, we have removed or altered any details that could potentially identify individuals and their family members. Given the potentially sensitive nature of discussing caregiving burdens, the interviewer remained attentive to participants’ emotional comfort during interviews, and participants could skip any question or take a break at any time. The study followed standard ethical guidelines for research with human subjects, as outlined in the Declaration of Helsinki.

## Results

Through the thematic analysis of the 14 interviews, we identified five major themes: (1) Caregiving as duty versus choice, (2) Challenges and burdens of caregiving, (3) Rewards and personal fulfilment, (4) Preference for family care over institutional options, and (5) Desire for support and integration into the care system. These themes and their sub-components encapsulate the caregivers’ perceptions and lived experiences. In the quotations below, participants are referred to by their assigned code (P1–P14).

### Theme 1: Caregiving as duty versus choice

In an attempt to summarise the reasons behind deciding to become an informal caregiver, even though each situation is unique and reasons are not mutually exclusive, we observed that a prominent theme was how caregivers situated their role on a spectrum from **obligation** to **voluntary choice**. Most participants described a strong sense of familial or moral duty to care for their loved one, even if they were not explicitly coerced. At the same time, many insisted that they willingly embraced this duty out of love. This nuanced interplay of obligation and devotion is illustrated by their accounts.

Several caregivers felt that taking on the responsibility was essentially *expected* of them as family members. They spoke of a norm that one “must” care for an ailing relative. As one daughter explained, *“it was my responsibility as a daughter… I was raised with the notion that parents must receive the care that their children owe them”* (P9). Having grown up with this value, she saw little question that she would care for her aging parents when they needed help. Similarly, a wife (P11) caring for her chronically ill husband viewed caregiving as a *univocal duty*: *“I believe it is a moral duty*,* first of all. And secondly*,* it’s part of being family.”* In her view, anyone who sees a family member in need should feel compelled to help. This moral imperative was echoed by others who described caring for parents as a way of repaying or honouring what their parents had done for them earlier in life. For instance, one woman (P6) noted that her elderly mother had cared for her in the past, *“so now it’s my turn to help – it’s my duty to return the favour.”*

Even though these caregivers used the language of obligation, they generally did not frame it as a burden imposed against their will, but rather as a reciprocal responsibility they accepted as just and necessary. One daughter-in-law (P14) similarly felt a profound responsibility toward her father-in-law, whom she began caring for after her mother-in-law passed away. She expressed deep empathy for his condition, stating “*I feel pity for him… he’s our elder… if you can care for a stranger*,* then surely you care for your own elder?*”. In her view, no family member in need should be left without care. This example underscores that compassion and moral duty extended beyond immediate parent-child bonds, motivating even in-laws to assume the caregiving role out of love and obligation.

In some cases, the lack of alternatives made the decision for them. A few participants described being *“constrained by the situation”* (P3) when no one else was available to provide care. One caregiver (P5) said plainly: *“There was no alternative in my case. This was it… at some point*,* you just have to do it.”* She felt she could not refuse the situation or leave it to someone else, because there was literally no other family nearby and formal care services were either unavailable or unaffordable. Another participant (P1), a former nurse, had initially not expected to become the sole carer for her mother but stepped in when her father’s health suddenly declined: *“Yes*,* I was forced by the situation. I didn’t know it would turn out this way.”* She went on to explain that while she had always been inclined to care for others (given her nursing background), the magnitude of her father’s needs ended up far greater than anticipated, effectively leaving her with no choice but to assume a full-time caregiver role. These narratives highlight that external circumstances (such as absence of other caregivers or unforeseen worsening of a loved one’s condition) often dictated the caregiving decision, independent of the caregiver’s initial wishes.

On the other end of the spectrum, many caregivers asserted that they did not feel *externally* obligated or coerced, but rather chose to care out of love and personal commitment. *“I never feel obligated and I never will*,* if it’s a member of my family. It’s my grandmother*,* she supported me so much*,* of course I’ll be there for her.”* (P10) said a granddaughter. Despite others assuming someone relatively young might feel tied down, she emphasized her close bond and willingness. Likewise, an adult son (P2) caring for his widowed father described stepping in simply because *“there was nobody else to help and I do it gladly.”* He said, *“No*,* not at all [did I feel obliged]. His health deteriorated and in the end there wasn’t really anyone else to care for him*,* so I help with all I can*,* gladly.”* Even participants who spoke of duty often simultaneously said that their motivation was rooted in affection. They stressed that they *wanted* to ensure their relative was cared for properly. This suggested that *obligation* and *choice* are not mutually exclusive in the caregivers’ minds – many saw caregiving as something they ought to do and something they freely chose to do out of love.

Therefore, cultural and familial expectations strongly influenced the attitudes and behaviours of caregivers in our sample, instilling a sense of duty to care for their aging relatives. Most participants internalised this duty, viewing it as the right thing to do rather than an unwanted imposition. Some were effectively compelled by circumstance when no alternatives existed, whereas others felt entirely self-motivated. Even those who felt “no choice” often described ultimately embracing the role. This theme underscores that caregiving in this context is rarely seen as a purely voluntary leisure activity; it is linked with norms of family responsibility. Yet the caregivers’ willingness to carry that responsibility, often with pride, is equally evident in their reflections.

### Theme 2: Challenges and burdens of caregiving

Participants often candidly described numerous challenges in their day-to-day caregiving experience. The burden of care manifested in physical, emotional, social, and financial dimensions of their lives. Many caregivers were stretched trying to meet the needs of their relative while also managing other responsibilities, often at the expense of their own health or leisure. Four sub-themes captured these burdens: physical strain, emotional stress and mental health, impact on personal life (social and occupational), and lack of support.

Physical strain was a common thread, especially for those caring for relatives with significant mobility impairments or heavy care needs. Tasks such as lifting, bathing, or turning the person in bed demanded considerable strength and endurance. One daughter (P1) caring for a father who had become bedridden shared how exhausting the daily routine could become: *“All day you have a schedule with him… you can’t really relax… you sit down for a moment and it feels like as soon as you do*,* you have to get up again to lift him”.* She described having to constantly be on her feet – feeding him, changing him, helping him reposition – to the point of near physical exhaustion. Another caregiver (P11) noted the toll of disturbed sleep, as she often had to attend to her husband’s medical needs at night: *“Sometimes if I am overtired and haven’t slept*,* it all becomes very hard to manage”.* The physically demanding nature of caregiving left several participants chronically fatigued. As one put it, “*physical and mental exhaustion appears*,* this state of being constantly unrested”* (P10). Indeed, one caregiver (P14) who had recently undergone back surgery found lifting and repositioning her bedridden father-in-law especially arduous, as it often aggravated her own pain. Yet she persisted out of a sense of duty, explaining that *“I’m not healthy either… but well*,* what can you do*,* he’s old… I feel sorry for him.”* This illustrates how caregivers frequently push through physical hardship - and even risk their own health - due to empathy and commitment to their loved ones.

Closely intertwined was the emotional and psychological stress of caregiving. Participants spoke of feelings of worry, anxiety, and being overwhelmed. Many were constantly on high alert about their loved one’s condition, which created mental strain. For example, P11 recounted experiencing a panic attack during a pandemic lockdown when she felt entirely alone in managing her husband’s care: *“It was very*,* very hard… for me it was overwhelming”.* Even outside of acute crises, the ongoing responsibility weighed on caregivers’ minds. They described stressors such as seeing the care recipient’s health decline, dealing with difficult behaviours (in the case of dementia, for instance), or simply the relentlessness of caregiving routines. For example, one participant (P13) caring for her grandmother with dementia described having to devise creative strategies to manage difficult behaviours. She learned to *“get down to her level… I did all sorts of antics with her*,*”* turning daily care tasks into games or music to keep her grandmother calm. These tactics – developed through personal research and even consulting a doctor for advice – prevented agitation but required enormous patience and emotional stamina. This example highlights the extra emotional labour involved in caring for a cognitively impaired loved one, adding another layer to the usual levels of stress that caregivers face.

P10, who balanced a job with caring for her grandmother, explained how stress accumulated: *“With the stress and the large workload*,* exhaustion sets in… and eventually frustration comes in as well”.* This highlights how burnout can emerge: one feels drained and frustrated, perhaps due to lack of rest or lack of progress in the loved one’s condition. A few caregivers admitted to moments of irritability or despair. *“It’s very hard for me when I’m also overworked with tasks and I have my own emotional baggage”* (P11) – she noted, acknowledging that her own emotional state sometimes made coping harder. Notably, however, even when describing such low points, caregivers were quick to temper them with their commitment (often saying, in effect, *“it’s hard*,* but I carry on*”). Nonetheless, it is evident that mental health strains were present, and participants recognised that caregiving had affected their psychological well-being (even if few had time to address it). One younger caregiver (P10) observed, *“No one really looks after our mental health… aside from us trying to encourage each other”.* This remark demonstrates that caregivers felt largely left to manage their stress on their own, without formal outlets or support for their emotional needs.

Another significant challenge was the impact on personal life, including social relationships and work. Caregivers often had to sacrifice hobbies, social activities, or even employment opportunities due to their responsibilities. Several participants described leading a much more restricted life since becoming a caregiver. *“Our life – we used to be very socially active – we had to retreat into a great quiet.”* (P11) - this participant recounted how after her husband’s cardiac events, their previous social circle faded away and they became largely homebound, with friends seldom visiting. The sense of isolation was noted by others as well: they could not easily leave the house or had to turn down invitations because of caregiving duties. In terms of work impact, one of the most striking examples was P11’s experience: *“At that time I had to give up my job for a year*,* and I broke off from my career a bit”.* She stated that a year away from work set her back professionally. Another caregiver (P5) reduced her working hours to part-time to accommodate her father’s care, resulting in reduced income and stalled career progression.

The financial consequences were tied to this: not only did some lose earnings due to cutting work hours or quitting jobs, but many also incurred extra expenses for the needs of the care recipient. Medications, medical supplies, home modifications (for example special beds or wheelchairs), and private medical consultations were frequently mentioned costs. For instance, P11 recalled paying out-of-pocket for a life-saving stent for her husband which costs the equivalent of three of her monthly salaries. Such expenses put strain on family finances. Altogether, caregivers often endured a combination of social sacrifices and economic hardships, which added to their stress.

The fourth sub-theme was the profound lack of external support that caregivers experienced, amplifying all the other burdens. In most cases, participants, to different degrees, felt they had little to no help from formal services or sometimes even from other family members. Many were sole caregivers. *“No help from anyone. Only me and my family.”* (P11) - this participant summarised what others echoed: aside from perhaps a spouse or sibling occasionally assisting, they received no formal respite or regular relief. A few hired paid helpers briefly but found it unsatisfactory or too expensive. P8 tried to find someone to care for her father-in-law during work hours but noted that *“you can’t find anyone [to hire] and the nursing home is expensive*,*”* so she had to manage together with her husband. Interactions with the health and social care system were often described as frustrating.

Participants complained about poor communication from healthcare providers and feeling that the system largely ignored them as carers. As one caregiver (P10) put it bluntly, *“Our healthcare system is really bad at communication… they don’t talk [to us]”.* She recounted instances where she struggled to get information from hospital staff about her grandmother’s condition, leaving her feeling helpless and anxious. Another caregiver (P7) observed that informal carers *should* be more involved in patient care because it benefits the patient’s comfort, yet in practice, hospitals did little to include or inform family: *“[Family] should be involved a great deal… for the patient’s psychological comfort… but they aren’t”.* The general sentiment was that formal services were either absent or unhelpful, forcing caregivers to rely on themselves. This lack of support exacerbated other challenges, as caregivers had nowhere to turn for relief or guidance. Some caregivers did mention support from friends or other kind individuals (such as a neighbour who was a nurse, or a doctor who gave advice), but these were ad-hoc and informal. Participants were not aware of any organised caregiver support groups, and the burden of care was therefore almost entirely on the individual and their immediate family.

Overall, this theme shows a picture of caregivers who were overburdened and under-supported. They faced intense physical labour, health risks, emotional strain bordering on burnout, significant life changes in terms of social isolation and work disruption, and they did so with minimal assistance.

### Theme 3: Rewards and personal fulfilment

Despite the difficulties, many caregivers identified personal rewards or fulfilling aspects of their caregiving experience. After mentioning the hardships, most of them were able to name positive elements – whether emotional, relational, or spiritual – that gave them a sense of purpose and motivation. This theme reflects how caregivers derived meaning and sometimes mutual benefit from their role, experiencing caregiving not only as burden but also as a source of satisfaction, growth, and strengthened relationships.

One of the most commonly mentioned rewards was the emotional satisfaction of helping a loved one. Caregivers took pride in being there for their family member and ensuring they were well cared for. *“I’m glad that I can help”* (P1) said a son about caring for his father. He described feeling good that he could “be a support” for his elderly father and make his father’s life a bit easier. This sense of purpose – knowing that one’s efforts make a tangible difference in the loved one’s quality of life – was highly valued. A daughter (P6) said that for her and her sister, keeping their mother at home with family was deeply meaningful: *“I never even considered not being there for my parents.”* For her, the reward was in the very act of *being there*, fulfilling a role that she considered extremely important. Several participants noted that caring for someone who once cared for them (parent or grandparent) felt like giving back and came with a sense of personal fulfilment and honour. As P7 mentioned, *“He raised me… he cared for me*,* and it was never a question that I would care for him [when he needed it].”* In this statement, we observe not only duty but also a kind of *emotional reciprocity* that was rewarding to the caregiver; P7 felt happy to return the kindness and love her grandfather had shown her.

Many caregivers also spoke about the positive feelings they experienced when they saw their loved one comfortable or improving because of their efforts. Even small wins – such as managing to calm the person’s anxiety, or seeing them recover from a health setback, gave the caregivers encouragement. Caregivers often celebrated the victories, however small, and these moments alleviated their stress and reinforced their commitment.

Caregiving also seemed to strengthen family bonds for some. Spending so much time together, though challenging, sometimes deepened the emotional connection between caregiver and care recipient. One wife (P11) reflected that through caring for her husband during his illness, their relationship had gained a new dimension of closeness and appreciation for time together. She said she cherished the quiet moments they spent each day, even if it was just sharing stories or holding hands – those moments felt like *“extending someone’s life in a meaningful way”* and a pleasure to be able to do. Similarly, a granddaughter (P10) mentioned that caring for her grandmother brought them closer: the two shared many conversations and memories during care routines, which the granddaughter found rewarding as it enriched her understanding of her grandmother’s life and wisdom. In these ways, caregiving acted as a bonding experience, allowing for intimacy and gratitude.

Several participants even spoke of personal growth and resilience gained through caregiving. They were able to learn new skills (medical tasks, navigating healthcare bureaucracy, etc.) and discovered inner strength. P3 said she learnt patience and how to cope with difficult situations, while P2 mentioned that succeeding in tasks that seemed initially intimidating came with a feeling of accomplishment. Another caregiver emphasised the spiritual gratification she experienced from helping her loved one. Reflecting on her motivation, she said: *“as long as I can*,* as long as the good Lord gives me strength… why not do good?”* (P12). Even though she admitted not knowing who would care for her in old age, she viewed caregiving as a calling to “do good” while she was able to. Personal faith and a sense of purpose made the caregiving role rewarding for her, reinforcing the fact that many caregivers found meaning and fulfilment in their efforts.

Importantly, most caregivers described a mix of positive and negative feelings, often in a cyclical manner. As one participant (P1) summarised: *“You have great moments of satisfaction*,* of fulfilment*,* you feel gratified*,* after which fatigue sets in… and frustration”.* The rewarding moments – seeing their loved one smile, receiving a heartfelt “thank you,” achieving a small health improvement – gave them *“highs”* of fulfilment that kept them going. Then the exhaustion and stress would catch up, leading to lows of frustration or doubt. But still, the positive sense of doing the right thing and the love for their family member pulled them back.

Although difficult, caregiving was seen as a meaningful and rewarding act. Caregivers gained personal satisfaction from helping their loved ones, felt a sense of purpose, strengthened family bonds, and often experienced personal growth. These positive aspects seemed to serve as protective factors against burnout – they were the emotional rewards that kept up the caregiver’s motivation. Recognising these rewards is important, as it highlights that caregivers were not only victims of burden but that they can also find genuine value in what they did.

### Theme 4: Preference for family care over institutional options

When discussing LTC arrangements, participants expressed a strong preference for family-based care and a reluctance to use institutional care (such as nursing homes or residential facilities) for their loved ones. This was shaped by cultural norms, personal experiences, and perceptions of quality and cost. In essence, “home is best” was the prevailing sentiment, with institutionalisation seen as a last resort if no other solution is viable.

Many caregivers shared the view that being cared for by family in one’s own home (or the home of a relative) was better for the older person’s well-being. They believed that familiar surroundings and loved ones’ presence provided comfort that cannot be matched by an institution. One participant (P7) said she never seriously considered a care home for her grandfather because *“I think his condition would have worsened a lot if he were left in a hospital or nursing home with unknown people.”* She felt that the psychological benefit of being with family (speaking the same language, sharing memories, receiving personalised attention) was crucial to maintaining his health and spirit. This belief in the therapeutic value of family care was common. Another caregiver (P8) noted that when her father-in-law was hospitalised briefly, he became depressed and disoriented, which affirmed to her that *“he does much better at home with us around.”* In their eyes, a care facility could not provide the same level of individualized care or emotional support as a devoted family member.

Cultural values around duty and attachment also played a role in their reluctance to institutionalise their loved one. Several caregivers described a stigma to the idea of “putting” a parent in a nursing home. As one described, *“to take your mother to a home seems… too much; to be cared for by a stranger and be like you have no one*,* when you know you have children [is not right]”* (P3). She remembered that her mother had once suggested *“maybe I should go to a nursing home*,* so I don’t burden you*,*”* but she found the idea unacceptable and even painful. This perspective is deeply rooted in Romanian familial norms where children feel obligated to personally ensure their parents’ care. Another participant (P6) shared a similar story: his mother, while still healthy, once mentioned in passing that if she ever became a burden, perhaps a care home could be an option, but he and his sister immediately jumped in and said: *“we’ll take you in with one of us [if it comes to that]”*. They assured their mother that she would live with family, not among strangers, highlighting a strong family commitment to care inside the family, partly out of love and partly due to the social expectation that *“good children”* do not send their elders away.

Pragmatic concerns about quality and trust in institutions also underlay their preferences. Some caregivers doubted that nursing homes could provide adequate care, citing extremely aversive experiences or negative perceptions of such places. P7 described it as very hard to imagine her grandfather in a facility, suggesting she doubted he would get the attention he needs. There was a general mistrust that strangers or overworked staff would care for their loved one with the same diligence and affection as family would. P3 mentioned: *“To be wiped/cleaned by a stranger… when you know you have children [who could do it]”* as an undignified scenario they wanted to avoid for their relative. This indicated a concern for the personal dignity of the care recipient: being among family was seen as more respectful and loving, whereas being attended by strangers might feel degrading or lonely for the elder. Additionally, a few participants referenced generally poor conditions or understaffing in public elderly care institutions in Romania, which reinforced their unwillingness to consider that route. Even without firsthand experience, the bad reputation (also in light of recent public scandals of the conditions in some) of elderly care homes made family care the preferred choice.

Another factor was of financial nature: several caregivers pointed out that institutional care, especially any decent private facility, would be prohibitively expensive. *“The nursing home is quite expensive*,* nothing you can do about that.”* (P10) - she had inquired locally and found the costs far beyond what the family could afford on their combined salaries. For her father-in-law, they concluded it was financially more feasible to adjust their work schedules and care for him at home than to pay for a care home. Thus, even if she might have considered a facility, the economic barrier was high. Without state subsidies or affordable options, institutional care was effectively off the table for most of our participants.

It is important to note that while most of them preferred to continue family care, they were not necessarily dogmatic in their viewpoint; a few acknowledged that if circumstances worsened, an institution might become unavoidable. A participant (P4) who was caring for her mother admitted that she once considered placing her mother in a care home because she was struggling, but ultimately *“couldn’t afford it… and I felt I could do it myself instead”.* Interestingly, after saying that, she added ***“****Yes*,* why not [take her into my home]*,* that way I know she’s safer*,* I see her every day.”* This suggests that even when finances forced her hand, she found reassurance in the family care choice. For most, the idea of a care home was essentially the last resort if the caregiver became unable to provide care (for instance, if the caregiver’s own health failed). None of the participants had yet reached a point of considering it imminent, and some were determined never to let it happen.

These caregivers showed a clear preference for keeping the care of their elder within the family. They perceived family care as warmer, more trustworthy, and morally appropriate. Nursing homes were viewed with scepticism and often guilt, and were generally only contemplated in extreme situations. This attitude aligns with broader cultural norms in Romania that value family care and reflects practical concerns about the availability and quality of formal LTC services.

### Theme 5: Desire for support and integration into the care system

While the caregivers in this study preferred to keep their relatives out of institutions, they also expressed a strong desire for more support and better integration with formal health and social care systems. This theme highlights the expectations caregivers had regarding how informal carers like themselves and their care recipients should be treated and supported in Romania’s LTC landscape. Participants offered insights into improvements that could alleviate their burden, including better communication with healthcare providers, training and information, respite services, and formal recognition of their role. Their perspectives suggest that caregivers did not want to hand off their duties, but they wanted to be acknowledged, included, and assisted as part of the elder care ecosystem.

Notably, many caregivers reported receiving little to no help under the current system – a key reason their desire for support was so strong. One daughter-in-law (P14) said: *“I have no support… I have no help. I have nothing.”* Apart from basic medical care during her father-in-law’s brief hospitalisations, she and her husband managed all care themselves, with only occasional help from relatives. The almost complete absence of formal and community assistance explains why participants welcomed ideas such as caregiver training, relief care, and inclusion by healthcare providers. Integrating even modest support would, in their view, contributed to their ability to continue providing family care without feeling so alone and strained in this journey.

A recurring point was the need for improved communication and inclusion in healthcare settings. Many caregivers felt excluded or invisible when dealing with doctors, hospitals, or other services. They advocated for being treated as partners in the patient’s care. For example, P7 stated: *“Informal caregivers should be involved a great deal… for the patient’s psychological comfort”.* From her viewpoint, hospitals and clinics should recognise that having a family caregiver present or informed can benefit the patient, and thus they should facilitate that involvement (such as allowing the caregiver to stay with the patient or keeping them updated on medical plans). Another caregiver (P5) noted a positive experience where a doctor taught her how to perform certain home care tasks for her father, which she found empowering. She suggested this should be standard: *“it’s very important… that doctors explain to us what we need to do and how [to do it]”.* In contrast, others had negative experiences of being ignored or not instructed, which left them anxious and unprepared. Therefore, a clear expectation was that the healthcare system should systematically engage and empower caregivers – through better communication, providing guidance, and taking their insights into account since family carers often know the patient’s day-to-day condition best.

Another widely mentioned support need was access to information and training. Caregivers often felt they had to teach themselves how to perform various actions and become effective carers. They said that some formal training or educational resources would be helpful, especially for complex care tasks or managing specific illnesses. One participant (P1) proposed that there should be *“courses in every city… [for caregivers] to learn what to do”*. She imagined a situation where local authorities or health services offer basic caregiving workshops on topics such as elderly mobility care, managing medications, recognising warning signs, assessing risks, etc. This idea resonated with others as well, as many had struggled through trial and error or internet searches to find information. P11 also emphasized learning needs, saying that families of chronic patients should be thoroughly educated about what problems to expect and how to handle them. In essence, caregivers wanted the system to equip them with knowledge, rather than leaving them finding their way in the dark. Some noted that even a simple informational brochure or a designated nurse to answer questions would be a step forward.

Respite and practical support services were another expectation, even though caregivers doubted there was anything currently available. When asked what could improve their day-to-day caregiving, some participants hesitantly mentioned that having someone to relieve them occasionally would be a significant help. P9, for example, wished there were volunteer programmes that could offer specialised assistance and therefore offer her some valuable help and relief. Other caregivers (P3) said it would be ideal if the local social services could send a carer a few times a week to help with heavy tasks such as bathing, or just to give them some time off. The common theme was a need for temporary relief: time to rest, run personal errands, or simply recharge, knowing that their loved one is safe with a competent and empathic helper.

Financial support or incentives were also touched upon. A couple of caregivers were aware that in some countries, family caregivers receive stipends, or the care recipient gets an allowance that can indirectly support the caregiver. In Romania, implementing such a law is still in its very early stage, and caregivers had no one to turn to for help with understanding how to benefit from it [12]. P7 remarked that she gave up some paid work and that *“a financial help from the government would be important.”* While money was not the primary motivator, easing the financial burden would certainly reduce stress (e.g., to hire a helper occasionally or purchase better supplies). The expectation here was that policymakers should recognise the economic value of informal care and compensate it in some form or at least subsidise the costs. This was not a dominant theme in the narratives, possibly because participants had little hope it would happen, but it was mentioned as part of an ideal support scenario.

In sum, while Romanian informal caregivers in this study showed remarkable self-reliance, they also articulated clear expectations for a more carer-friendly system. They wanted to be seen as partners by professionals, to receive guidance and training, and to have occasional respite and perhaps financial aid. These desired supports did not replace their family-centric approach – instead, they would offer the opportunity for them to continue providing care at home, aligning with their preferences.

## Discussion

Our study provides an in-depth exploration of the perspectives and experience of informal caregivers of older adults in Romania, revealing how they navigate the caregiving role in a context of high familial expectations and low formal support. The research that we conducted adds to the extremely scarce qualitative evidence on Romanian informal carers’ reflections on their work, role, meaning and attitudes towards LTC in the larger healthcare system during a critical time for the future of home care. Our findings highlight a complex interplay between cultural norms, personal motivations, and systemic challenges, offering insights relevant to health services and policy.

One major finding is the powerful influence of familial and cultural duty on caregivers’ decision to assume and continue their role. Participants often framed caregiving as a “moral obligation” or a natural part of being family, echoing traditional norms in Romanian society where children are expected to care for elderly parents [5]. Romania’s care regime is strongly familialistic, with social norms dictating that families (particularly women in the family) shoulder elder care responsibilities​. Indeed, most informal caregivers in Romania are wives or daughters, reflecting these gendered expectations ​ [14]. Our evidence adds to these observations: caregivers internalise these norms to the extent that they rarely perceive caregiving as a true choice. Even when feeling “constrained by the situation”, they often come to embrace the role as the right or only thing to do as a family member. This sense of duty can be double-edged – on one hand, it provides caregivers with a clear purpose and resolve, but on the other, it may lead them to neglect their own boundaries or hesitate to seek help. Indeed, none of our participants readily considered fully relying on formal care services, which speaks to how deeply ingrained the familial care ethic is.

In addition, caregivers in our study also asserted their personal affection as key motivator. Many did not feel “forced” by family members; rather, they were driven by love, gratitude, and reciprocity. This resonates with findings in other contexts that caregiving is often a mix of obligation and desire – what scholars have termed *“mixed motivation”*. For example, a study of family carers in a different European country described caregiving as something people *both feel they must do and want to do*, out of love and duty simultaneously​ [2]. Our findings can be interpreted also in the light of the theory of gift proposed by Marcel Mauss [31] that defines gift as not only a mere material or economic exchange, but also as a relational and social exchange that determines roles and status in the context of the exchange. In our case, when a caregiver donates their time and energy to a loved one, they are reinforcing their relationship and receiving in return motivation and satisfaction. At the same time, they are affirming a specific role and status in the community (i.e. being a caregiver). Our findings reinforce this narrative in the Romanian context. Recognising this is important for policy and practice: interventions to support caregivers should validate the emotional rewards and values they attach to caregiving while also addressing the fact that duty alone can overburden individuals when external support is absent.

The challenges and burdens reported by our participants are aligned with the well-documented strain on informal carers, but with specific manifestations given Romania’s context. Caregivers experienced physical exhaustion, emotional stress, social isolation, and financial difficulties​. However, these issues may be intensified in Romania due to the absence of formal services and support. For instance, many European countries already do or plan to provide some form of respite care, training, or financial allowances to caregivers, whereas Romania offers virtually none [9]. ​ The caregivers in our study had to improvise solutions and often felt completely alone in their role. Consequently, Romanian caregivers may be at heightened risk of burnout and negative health outcomes. Indeed, some of our participants described symptoms of burnout (e.g., chronic fatigue, panic attacks). This aligns with broader evidence that intensive informal caregiving – particularly without respite – is associated with higher rates of depression, anxiety, and health problems in carers​ [2]. The lack of support theme in our results clearly illustrates how the Romanian system’s gaps leave carers to carry a great load, often alone.

On the positive side, our participants also found meaning and personal rewards in caregiving, which can buffer stress. The reported feelings of fulfilment, pride, and strengthened relationships they reported are examples of what is sometimes called *“caregiver gain”* in the research literature [32]. Studies in other countries have noted that some caregivers experience personal growth, find a new sense of purpose, or feel satisfaction from keeping loved ones at home in comfort​ [33–35]. In Romania, these positive aspects might be especially important given that external validation or reward (for example financial compensation) is absent; therefore, the intrinsic rewards become a crucial sustaining force. Our findings suggest that many Romanian caregivers derive their identity and self-worth in part from the caregiving role, seeing it as an expression of love and duty fulfilled. This has implications for how any future interventions are framed – acknowledging caregivers’ pride and commitment, and perhaps building on it (for example, by offering ways to make caregiving easier rather than replacing it).

Another relevant aspect of our study is the insight into caregivers’ attitudes toward formal long-term care. Most of the participants preferred family care and were less keen to rely on institutional care. This preference is not surprising in light of Romania’s history and current LTC infrastructure. Elder care facilities in Romania have been few, often of uneven quality, and carry a social stigma. The low utilization of nursing homes (only ~ 0.4% of older people are in residential care​) is both a cause and effect of the dominance of family care. Our findings shed some light on the reasons behind this low uptake: cultural expectations, negative perceptions of quality, and high costs. The mention that “the nursing home is expensive” and simply not an option for some, aligns with policy analyses showing that formal LTC services are severely undersized in Romania and often unaffordable for the average family​ [9]. Consequently, even if caregivers are overwhelmed, placing a relative in a nursing home is generally off the table, unless absolutely unavoidable. In Romania the sense of guilt or failure associated with institutionalising a parent appears strong. From a policy perspective, this suggests that simplistic solutions (e.g. enhancing capacity through building more nursing homes) will not automatically solve the elderly care issue. Indeed, families may not use them unless there is an alliance ensuring that using such solutions does not mean abandoning their loved one, and that certain quality and accessibility standards are met. Instead, emphasis should be put on strengthening in-home and community care services, which align with caregivers’ preferences to keep elders at home.

The caregivers’ need for support points to clear directions for improving the health and social care system. They essentially outlined a wish for a more “carer-friendly” environment: better communication from professionals, inclusion in decision-making, access to training, respite options, and perhaps financial or employment-related support. These wishes are very much in line with recommendations from international bodies on supporting informal carers [2]. Romania, as an EU member, is encouraged under this strategy to develop a more supportive policy framework for carers. Our findings provide evidence that Romanian caregivers would welcome such changes. In particular, introducing training programmes or informational resources for caregivers could be a relatively low-cost, high-impact intervention. Another area of intervention is respite care. Although currently extremely limited, even incremental steps (such as day care centres or occasional in-home respite via community nurses) could substantially reduce caregiver strain. Given that Romania devotes almost all LTC funding to modest cash benefits for disabled persons and almost nothing to services​ [9], a rebalancing toward services could directly address some of these needs.

Importantly, the discussion of support does not imply that caregivers want to give up part of their role – rather, they want to perform it better and with less personal cost. This nuance is vital - policies should not aim to replace family care (which caregivers and elders prefer), but to support and supplement it. The integration of informal carers into care teams – for instance, involving them in hospital discharge planning or chronic care management – would be a good practical step. Studies from other countries show that when caregivers are well-informed and included, patient outcomes improve and caregiver stress can be reduced through greater confidence and clarity of role [36]. In our study, caregivers explicitly asked for inclusion and guidance, indicating their willingness to collaborate with formal services if given the chance.

When interpreting these findings, it’s important to note the context: Romania’s demographics and social changes may test the sustainability of the informal care model. The country’s population is aging and shrinking due to low birth rates and high emigration, projecting the 2024 old-age to working-age ratio of 35% to become 60% by 2060​ [37, 38]. Many working-age Romanians (often women) have migrated to Western Europe as paid caregivers or for other work; indeed, the Romanian emigrant population in the OECD grew faster than any other major origin country (growth rate of 200% between 2000 and 2015) and it currently accounts for more than 4 million people, a share of around 20% of the total population [38]. This lead to a “care drain” phenomenon where fewer family members remain to care for elders at home [39, 40]. Some participants in our study mentioned this, describing siblings abroad or difficulty finding anyone to hire locally.

This trend, combined with increasing life expectancy and chronic illness, means the demand for elder care is rising while the pool of traditional caregivers may be shrinking. Some caregivers already felt they had “no alternative” because other family members were not present. The reliance on a single caregiver (often an older spouse or one adult child) could intensify. This points to an urgent need for Romania to invest in formal LTC infrastructure and caregiver support or risk a scenario where frail older adults have neither sufficient family nor formal care. Our findings come at a time when policy attention to LTC in Romania is reportedly increasing – for example, inputs for a national LTC strategy have been developed​ [41]. These findings can inform such efforts by centring the voices of caregivers.

To summarise, our study confirms that Romanian informal caregivers operate in a challenging environment of high expectation and low support, but they demonstrate resilience and dedication rooted in cultural norms of familial care. They are asking for help not to abandon their role, but to continue performing it in a sustainable way. The results suggest that relatively straightforward measures – education, respite, inclusion in healthcare – could significantly improve caregivers’ experiences and outcomes. Furthermore, acknowledging and supporting informal carers is not merely a carers’ issue; it is integral to improving elder care outcomes. In countries like Romania where informal carers provide the vast majority of care​, any health services reform or aging policy must treat them as key stakeholders. Supporting caregivers will ultimately benefit the older persons they care for, through more competent care and caregivers who are less exhausted and more able to cope.

Our findings also point to several areas where further research is needed. First, future studies could explore in greater depth how different subgroups of caregivers - such as male carers, rural versus urban families, or older spousal caregivers - experience and negotiate care responsibilities in Romania, as these groups may face distinct challenges and resource constraints. Second, longitudinal research is warranted to examine how caregiving motivations, burden, and coping strategies evolve over time, especially as Romania’s demographic changes and labour migration trends alter family structures. Third, there is a need for implementation and evaluation studies testing specific support interventions (e.g. caregiver training, respite services, or structured collaboration between caregivers and health professionals) to determine which models are feasible and effective in the Romanian context. Comparative research across Eastern European countries with similar familialistic care regimes could further illuminate how cultural norms interact with different policy arrangements. Finally, future work should also investigate the perspectives of older adults themselves, to better understand their preferences, expectations, and experiences of receiving care at home.

## Conclusion

Caregivers in our sample largely perceive caring for an elderly loved one as both a duty and a personal act of love – a dual motivation that keeps them committed despite considerable difficulties. They face multiple challenges: heavy physical workloads, emotional and psychological stress, sacrifices in their social and work lives, and an absence of formal support systems. Yet, they also find profound rewards in caregiving, deriving meaning, satisfaction, and strengthened bonds with those they care for.

Interestingly, caregivers showed a clear preference for keeping care within the family and the home, avoiding institutionalisation of their relatives unless absolutely necessary. At the same time, they want to be integrated as partners in patient care, to receive guidance, and to have access to services that ease their burden.

The implications of our findings for health services and policy in Romania are significant. Policymakers should consider measures such as caregiver education programmes, respite care services, and potentially financial or legal provisions (e.g. caregiving leave or stipends) to support those who are caring for elders. In addition, strengthening home and community-based care services, would directly support caregivers’ preference to keep their loved ones at home and could delay or prevent the need for institutional care. In addition, improving communication and coordination between healthcare providers and family carers can be implemented also through training healthcare staff to engage with caregivers and creating protocols for including caregivers in discharge planning and care consultations.

For healthcare professionals, the message is to recognise the informal caregiver as an integral part of the patient’s care team. Moreover, interventions such as caregiver support groups (even informally organised) could be introduced to fight isolation and allow peer exchange of coping strategies.

The dedication of Romanian caregivers, as evidenced in this study, is a valuable asset to the society – it is the backbone keeping many older individuals supported in the community. However, relying on duty and goodwill alone is not sustainable as demographics shift. A more *caregiver-centric approach* is needed, where informal carers are not assumed to be an infinite resource but are actively supported and cared for themselves. This study’s insights can guide the development of policies and services that acknowledge caregivers’ contributions and meet their needs. Doing so is not only a matter of caregiver welfare, but a pragmatic strategy to ensure that elders receive quality care in the setting they overwhelmingly prefer – their own homes.

## Limitations

This study has some limitations that should be acknowledged when interpreting the results. First, the sample size (14 caregivers) is limited and, like all qualitative research, was not intended to be representative for the population of Romanian informal caregivers. The participants were selected purposively and were individuals who agreed to talk about their experiences; it is possible they are more open or resilient than caregivers who declined participation. Those struggling with extreme burnout or those who felt great guilt or resentment might have been less likely to volunteer, potentially biasing our sample towards more adaptive caregivers.

Second, interviews were conducted in Romanian, and the analysis was done in Romanian, with quotes later translated to English. While we took care to ensure accurate translation, some nuances or emotive expressions may not carry over perfectly, as translation is always an interpretive act.

Third, the study relies on self-reported experiences and perceptions. We did not interview the care recipients or other family members, which means we have the caregivers’ subjective viewpoint; there are aspects of the caregiving situation (such as quality of care provided or the elder’s perspective) that are not captured here, and which might have been useful in depicting a more comprehensive perspective.

Another limitation is that the study did not include caregivers who had already placed their relative in a nursing home or those who had stopped caregiving, which could provide a contrasting perspective on the decision to institutionalise or cease caregiving. Our focus was on current caregivers, so we primarily captured the voices of those actively managing at home. Additionally, the geographic context of participants, while mixed, was limited to one region of Romania (due to recruitment constraints). Caregivers in different counties or in very remote rural areas might face different challenges (for example, even poorer access to healthcare, or different community support networks) that were not fully represented.

It should also be noted that the data were collected in a time when Romania’s LTC policy landscape is evolving; any new policies or services introduced after the time of study (for instance, pilot programmes for respite care or the impact of the European Care Strategy initiatives) would not be reflected in participants’ experiences. Thus, our findings are a snapshot of caregiver experiences in an emerging period and policy environment.

Another potential limitation relates to the risk of allowing interview questions to shape or predetermine the themes [30]. We were attentive to this issue throughout the analytic process and approached coding and theme development inductively, constructing themes from patterns of meaning across the dataset rather than mirroring the structure of the interview guide. Nonetheless, because the interview questions touched on central aspects of caregivers’ experiences (e.g., motivations, challenges, support needs), there is an inherent possibility of some conceptual overlap between the topics explored in the interviews and the themes identified. Another limitation concerns broader challenges associated with reflexive thematic analysis, such as the interpretive role of the researcher. While reflexivity and team discussions were used to enhance analytic rigour, the findings remain shaped by the researchers’ interpretive judgments, which should be taken into account when considering transferability to other contexts.

Finally, there is the issue of social desirability bias. Given the strong cultural norms around family responsibility, participants might have been inclined to portray themselves as devoted and coping, perhaps downplaying feelings of anger, regret, or wishing they didn’t have to care, all emotions that can be taboo to admit. We tried to create a comfortable, non-judgmental interview atmosphere, and some did share frustrations and “negative” feelings, but it’s possible that not all such feelings were voiced. Further research might complement these findings with surveys or instruments that measure caregiver strain and well-being more quantitatively to see how those align with the narratives.

Despite these limitations, the study provides valuable qualitative insights. It complements the existing quantitative and policy data on Romania’s LTC system, and the consistency of themes across diverse participants suggests a degree of transferability to similar contexts within the country. Nevertheless, caution is needed in generalising the results to most Romanian caregivers. Each caregiving situation is unique, and factors such as the caregiver’s relationship to the care recipient, the specific illness involved, and available extended family support can lead to different experiences. Further research could expand on this work by including a larger and more diverse sample of caregivers, as well as exploring perspectives of care recipients or healthcare providers in contact with informal carers.

In conclusion, while not without constraints, this study’s approach offers a rich understanding of Romanian informal caregivers’ experiences, which can inform both local practice and contribute to the wider literature on caregiving in familialistic care systems. The limitations identified point to areas for future inquiry and underscore the importance of contextual sensitivity when applying these findings to policy or programme development.

## Supplementary Information

Below is the link to the electronic supplementary material.

Supplementary Material 1

Supplementary Material 2

## Data Availability

The datasets used and/or analysed during the current study are available from the corresponding author (DAL) on reasonable request.

## Abbreviations

LTC: Long-term care
EU: European Union
