# Supplementary material for: Informal caregivers’ perspectives on caring for elderly people in Romania: a qualitative study
Source: BMC Health Serv Res. 2026 Jan 23;26:255. doi: 10.1186/s12913-026-14085-1 (PMC12911341; doi:10.1186/s12913-026-14085-1)
Supplement: Supplementary file 1 — Supplementary Material 1 [file 12913_2026_14085_MOESM1_ESM.docx]

**Supplementary file 1 - English translation of the interview guide**

General Information

1. Who do you care for (relative, neighbor, or someone else)?

2. How long have you been caring for an elderly person?

3. Do you share caregiving responsibilities with anyone?

4. How do you organize your caregiving activities—for example, do you do this alongside full-time or paid work?

Tasks Performed

5. Briefly describe your role as a caregiver and your typical daily tasks.

6. How do you feel about caring for an elderly person?

7. Do you feel you have adequate information to care for an elderly person? If not, what kind of information do you need?

Resilience in Healthcare Services

8. What works well in your daily caregiving? What are you good at, and why?

9. What contributes to high-quality care in your situation (what solutions help with daily care work)?

10. What types of adjustments/solutions/adaptations do you use to deal with challenges (e.g., lack of information), changes (e.g., patient deterioration), and variations (e.g., needs, support)?

Patient Safety

11. What adverse events or near misses occur commonly, and how do you try to manage them?

12. How do you prepare for challenging situations or crises (e.g., when the patient’s condition worsens)?

Mental Wellbeing

13. What challenges (e.g., technical, resource-related, workload, competence, etc.) do you face daily?

14. What stressors (e.g., emotional, ethical, time management, workload, etc.) affect your work?

Support

15. What kind of support do you receive from the government, authorities, health leaders, medical staff, family, friends, or caregiver organizations?

16. How should informal caregivers be involved in patient care? How can patient and caregiver involvement be improved in situations like yours?

17. Based on your experience, how do other stakeholders (e.g., local organizations or interest groups) contribute to high-quality care for the elderly?

Overall

18. Could you suggest three specific ways your current working conditions as a caregiver could be improved?

19. What led you to become your relative’s caregiver? Did you feel obligated? If so, to whom?

20. What alternatives did you have? Are you satisfied with your decision?

21. What do you see as your benefits as a caregiver? What brings you the most satisfaction?

22. What are the professional and social consequences of being your relative’s caregiver?

23. Is there anything else you would like to add?

Thank you for your time and effort!
