# Supplementary material for: Informal caregivers’ perspectives on caring for elderly people in Romania: a qualitative study
Source: BMC Health Serv Res. 2026 Jan 23;26:255. doi: 10.1186/s12913-026-14085-1 (PMC12911341; doi:10.1186/s12913-026-14085-1)
Supplement: Supplementary file 2 — Supplementary Material 2 [file 12913_2026_14085_MOESM2_ESM.docx]

**Supplementary file 2 - Codes and sub-codes used in the analysis**

**Theme 1: Caregiving as duty vs. choice**

**Sub-codes:**

- Cultural expectation / filial piety (e.g., *“raised to care for parents”*)
- Moral duty (e.g., *“a responsibility as a daughter”*)
- No real alternative / default assumption (e.g., *“no one else could do it”*)
- Voluntary commitment (e.g., *“I do it with love”*)
- Reciprocity (e.g., *“they cared for me, now it’s my turn”*)
- Religious/spiritual belief (e.g., caregiving as a God-given task)

**Theme 2: Challenges and burdens of caregiving**

**Sub-codes:**

- Physical fatigue (e.g., lifting, sleepless nights)
- Emotional exhaustion (e.g., anxiety, stress, panic)
- Social isolation (e.g., fewer friends, “quiet life now”)
- Work-life conflict (e.g., reduced hours, leaving job)
- Financial strain (e.g., cost of care, medical expenses)
- Lack of help / support (e.g., “only me and my family”)
- Burnout indicators (e.g., irritability, loss of patience)

**Theme 3: Rewards and personal fulfilment**

**Sub-codes:**

- Emotional satisfaction (e.g., *“glad I can help”*)
- Sense of purpose (e.g., *“doing the right thing”*)
- Strengthened family bonds (e.g., reconnecting through care)
- Personal growth (e.g., patience, resilience)
- Spiritual reward (e.g., moral elevation, “it uplifts you”)
- Mutual respect and gratitude (e.g., care recipient’s appreciation)

**Theme 4: Preference for family care over institutions**

**Sub-codes:**

- Mistrust in institutional care (e.g., fear of neglect, “too expensive”)
- Emotional benefits of home care (e.g., comfort, familiarity)
- Stigma or guilt around nursing homes (e.g., *“not what good children do”*)
- Cultural/familial identity (e.g., “part of who we are”)
- Conditional openness (e.g., “last resort only”)

**Theme 5: Desire for support and integration**

**Sub-codes:**

- Desire for training/education (e.g., how to manage medications, wound care)
- Inclusion in medical care decisions (e.g., *“they should talk to us too”*)
- Communication gaps with healthcare providers (e.g., *“they don’t inform us”*)
- Respite needs (e.g., *“just a couple of hours to rest”*)
- Financial aid expectations (e.g., caregiver allowance, state support)
